# Molecular Screening for Cervical Cancer

**DOI:** 10.3390/genes16091041

**Published:** 2025-09-02

**Authors:** Toni Ricardo Martins, José Eduardo Levi

**Affiliations:** 1 Programa de Pós Graduação em Imunologia Básica e Aplicada—PPGIBA, Universidade Federal do Amazonas, Manaus 14040-903, Brazil; toni.martins@ufam.edu.br; 2 Faculdade de Ciências Farmacêuticas da Universidade Federal do Amazonas, Manaus 14040-903, Brazil; 3 Medical Research Laboratory in Virology (LIM 52), Institute of Tropical Medicine, Faculty of Medicine, University of São Paulo, Rua Dr Eneas de Carvalho Aguiar 470, São Paulo 05403-000, Brazil

**Keywords:** cervical cancer, screening, human papillomavirus

## Abstract

**Background/Objectives**: Cervical cancer (CC), a highly prevalent female neoplasia, has been prevented through repeated cervicovaginal cytology, the so-called Pap test, across women’s lifespans. The now undebatable role of Human Papillomaviruses in the etiology of CC and the development of high-throughput automated molecular amplification diagnostic platforms is allowing for the replacement of the Pap test with HPV testing. The objective of this review is to contextualize the current strategies for cervical cancer screening using molecular assays. **Methods**: The many existing screening tools relying on molecular markers and their advantages and drawbacks are discussed. **Results**: Testing for oncogenic Human Papillomavirus DNA is presently the mainstay strategy for molecular screening, replacing cervicovaginal cytology. **Conclusions**: The presence of HPV-DNA is the most sensitive marker for cervical cancer and its precursor lesions. However, its adoption has led to an increase in the number of screening-positive subjects, generating extra demand for triage resources. New algorithms and technologies are fast being developed to address this need, moving toward risk-based management.

## 1. Introduction

Cervical cancer (CC) is now the fourth most incident female cancer, following breast, lung and colorectal cancer, respectively. According to the GLOBOCAN, there were 661,021 new cases in 2022 worldwide [1]. However, the burden of CC is much more significant in some areas of the world than others, partly due to behavioral differences but mostly due to inequities in access to preventive measures. For instance, in 2020, Eastern Africa had an age-adjusted incidence of 40.1 new cases/100,000 women, while Western Asia showed the lowest incidence of 4.1/100,000 [2]. 

Acquisition of anogenital Human Papillomaviruses (HPVs) almost always occurs through sexual contact. The age of first intercourse, number of sexual partners, having unprotected sex, being a sex worker and having a history of sexually transmitted infection are all variables that strongly correlate with the risk of CC, explained by the transmission of HPV via this route [3]. More than 90% of HPV infections are cleared by the host within 24 months [4]. The remaining 10% of individuals who for some reason are unable to eliminate the oncogenic HPV are at risk of further developing grade 2+ cervical intraepithelial neoplasia [5].

However, the transition from an initial persistent HPV infection to CC takes more than 15 years, being slow and gradual. It involves dysplastic infected epithelial cells forming a low-grade intraepithelial lesion (LSIL), which may progress to a high-grade intraepithelial lesion (HSIL) and then to CC. Regressions of LSILs are common, and HSILs more frequently regress or do not evolve than progress to a carcinoma. This well-known natural history provides several opportunities to prevent the development of true precursor lesions by removing them through surgical interventions. The identification of such lesions through massive screening has been undertaken by many countries, implementing the method developed and advocated for by Dr George Papanicolaou. The method, named the “Pap” test after Dr Papanicolaou, basically consists of sampling the cervix and endocervical canal with a spatula, brush or swab and smearing the sample on a glass slide, followed by staining and looking for dysplastic or neoplastic cells under a microscope [6]. In the 1950s, this method received an important endorsement from the American and Canadian Cancer Societies, leading to its gradual implementation across these countries, followed by several European countries, in the next decades. Countries that have been able to deploy this method with high population coverage and in which women are committed to undergoing screening periodically have drastically reduced the incidence of this malignancy. However, a comparison between results obtained in different countries that perform cytology screening shows quite variable rates of reduction [7]. Higher reduction rates are mainly associated with the female population’s adherence to screening, but also with other socioeconomic factors. A characteristic widely claimed to be necessary for a high reduction rate is having an organized program in contrast to opportunistic screening. Nonetheless, Gustafsson et al. did not find a direct correlation between the kind of CC screening program and its performance in terms of CC reduction [7]. It should be noted that the CC incidence has also decreased in countries that have not implemented any kind of screening, suggesting that other risk factors have had an effect in these populations. HPV being a sexually transmitted agent, it is quite expected that the sexual revolution that took place in the 1960s would have increased the incidence of HPV infection and CC. Therefore, it is likely that screening not only reduced the incidence of this neoplasia but also prevented an explosion in cases in the 1970s/1980s. Another important risk factor that may have influenced the decrease in CC is the number of parities per woman, since during pregnancy the transformation zone becomes more exposed in the ectocervix. A reduction in the parity rate of a population correlates with a reduction in the CC incidence [8]. 

Some technical advances were introduced to the cervical cytology method, including the development of better fixative agents, improved collection devices and semi-automation. The shift to liquid-based cytology (LBC) was a radical change which, unintentionally, facilitated the further adoption of molecular assays. LBC differs from conventional cytology by directly immersing exfoliated cervical cells into a liquid medium containing fixatives. This medium maintains the cells’ morphology while preserving nucleic acids. During the process of cell collection, red cells, microflora and mucus are eliminated, resulting in a thin layer that allows for better visualization of the cellular details [9]. In addition HPV DNA is well preserved in this solution, and this synergy constitute one of the basis of molecular screening, as discussed below. 

The observed reduction in the disease burden due to screening has proved in practice that CC is a preventable disease. In fact, it is, so far, the only human cancer that may be eradicated, through the adoption of screening in addition to immunization [10]. HPV vaccines make use of in vitro-generated virus-like particles (VLPs) consisting of highly expressed L1, the HPV major capsid protein, which is assembled to form VLPs. These VLPs have been shown to generate high levels of antibodies, and, at a longitudinal follow-up, to prevent HPV infection among young girls and boys [11]. Further, immunized populations have displayed a much lower incidence of cervical lesions and genital warts, since the vaccines also target low-risk HPVs (genotypes 6 and 11), the causative agents of condylomas [12]. 

The polymerase chain reaction (PCR) and other nucleic acid amplification methods (NAATs) started to be used in clinical practice at the end of the last century. Improvements in automation and reagents progressively allowed for the expansion of the NAAT portfolio, particularly regarding the diagnostics of infectious agents. Virology benefited from these advances since viral diagnostics was usually performed through serology, i.e., examining the presence of specific antibodies, or culture methods, with both approaches presenting some drawbacks due to cross-reactivity and non-culturable viruses, respectively. HPV testing started to b used by gynecologists to elucidate undetermined cytology results, mainly for smears classified as ASCs-US (Atypical Squamous Cells of Undetermined Significance); when classified as HPV+, they would require further investigation, whereas when they were negative, they were considered a false-positive Pap test result. Since HPV had been determined to be a necessary cause of CC and there was the possibility of developing high-throughput testing methods for it, experts carried out large cohort studies comparing the performance of HPV-DNA to that of cytology in screening settings. These studies unambiguously demonstrated higher sensitivity for HPV-DNA, in addition to other advantages such as the possibility of expanding the screening interval to 5 years due to this increased sensitivity and the slow evolution from HPV infection to cervical lesions, a higher throughput and objective analysis, in comparison to the subjectivity of the Pap test [13]. 

Owing to these major scientific and technological advances, the elimination of CC is now feasible. This has led the WHO to outline practical actions to achieve this [14]. 

By the year 2030, adhering countries must have achieved the following:

- A total of 90% of girls up to 15 years old must have been vaccinated.
- A total of 70% of women >25 years old must have been screened at least twice, at the ages of 35 and 45, using a high-performance test (NAAT).
- A total of 90% of women with precancer and cancer must have been appropriately treated.

By implementing these three measures, countries should reduce the incidence of CC to below 4/100,000, the threshold of elimination defined from a public health perspective.

## 2. Sensitivity or Specificity: The Permanent Diagnostic Dilemma

Screening for any condition is by definition an initial step where the aim is to detect all true-positive subjects, inevitably resulting in false positives. Molecular amplification methods fit very well in this diagnostic space since their most notable characteristic is their ability to detect minimal amounts of RNA or DNA templates, i.e., their high sensitivity. Testing for HPV DNA or RNA allows for the detection of almost all cervical cancers and precursor lesions, allowing for early intervention and, in many cases, a cure. The drawback basically comes from the fact that HPV is quite a common sexually transmitted agent. Moreover, the most oncogenic HPV type, HPV 16, is also one of the most prevalent HPV types among healthy women [15]. Typically, results from HPV-DNA screening reveal that 10–15% of women are infected with oncogenic HPV types, more specifically one of the twelve genotypes currently considered obligatory targets for CC screening assays: HPVs 16, 18, 31, 33, 35, 39, 45, 51, 52, 56, 58 and 59 [16]. In contrast, cytology screening results in approximately 5% abnormal smears [17]. Thus, when moving from cytology to HPV-based methods, the number of women referred for further tests triples [18], which most health systems cannot afford. On the other hand, cytology may suffer from low sensitivity, partly because its performance is highly dependent on preanalytical factors, with the sampling of the transformation zone being the most critical. As this is related to the training of the health professional obtaining the cervical smear, including their choice of collection device, the clinical sensitivity of the method is quite variable. In Brazil, a historical analysis of cytological specimens obtained between 2006 and 2013 showed that more than 50% of them did not represent the TZ [19]. Liquid cytology does not solve the TZ sampling issue but controls better for other preanalytical and analytical variables. Large randomized clinical trials comparing liquid-based cytology and HPV testing observed that liquid cytology missed about 30-40% of high-grade lesions, paving the way for the replacement of cytology with HPV-DNA/RNA detection [13]. The studies described above clearly showed that cytology fails to detect some CC cases, generally those at an early stage, and, very importantly, adenocarcinomas [13], which originate in the endocervix, an area that is more difficult to sample. Thus, it is clear that HPV assays provide excellent sensitivity but poor clinical specificity. It is worth emphasizing that HPV assays are indeed specific for HPV-DNA; they do not cross-react with non-oncogenic HPV genotypes or other agents that may be present in the genital tract. But most HPV infections are transient and present in healthy women with normal cytology. Before HPV testing came into place, women with an abnormal smear were referred to colposcopy. Most CC screening programs do not have the capacity to perform a colposcopy in the recommended time interval for the 10–15% of participants who will receive positive results if HPV screening is adopted. Therefore, the most commonly adopted strategy, as of today, is to perform cytology following an HPV-positive result and, of practical importance, with the same LBC sample. This strategy relies on the high clinical specificity shown by cytology [20]. Furthermore, it has been observed that when cytologists are aware of the HPV+ status of the sample, the rate of lesion detection is improved [21]. It is important to note that currently HPV molecular screening and downstream triage assays require that cervicovaginal cells are fixed and preserved after collection to avoid degradation of the target molecules. To achieve this, samples are always collected in liquid cytology media. This allows for the timely execution of the screening test and further analysis of HPV+ samples using the chosen triage method. 

The aim of this review is to describe and contextualize the CC screening and triage methods in use and those under development and validation. 

## 3. Molecular Screening 

So far, all new CC screening methods have targeted HPV nucleic acids. Historically, Hybrid Capture (HC) was the first assay to be translated into clinical use, mostly in the context mentioned above, to clarify dubious cytology results. Subsequently, it was also the first to be adopted in studies evaluating molecular screening vs. cytology, with convincing results. With the introduction of the polymerase chain reaction (PCR), assays relying on this have progressively replaced HC. HC lacks the use of an endogenous human gene as an internal control, which is understood as a fundamental component of any molecular amplification method. Based on the knowledge that neoplastic cells need to express mRNA coding for the E6 and E7 viral oncogenes, methods detecting their presence would theoretically have higher specificity for grade 2+ cervical intraepithelial neoplasias (CINs), the true precursors of CC, eliminating HPV-DNA+/mRNA- samples from triage. In practice, methods targeting mRNA have shown clinical performance similar to that of HPV-DNA assays [22]. 

Nowadays, there are at least 48 countries using HPV assays as their primary screening tool [23]. Moreover, HPV molecular screening has been demonstrated to be cost-effective in all distinct socioeconomic settings where it has been properly evaluated [24,25]. Consequently, when HPV screening is adopted, it provides significant savings for health systems.

The market for CC screening is significant, in principle constituting all women between 30 (or 25) and 65 years old on Earth, comprising approximately 1.8 billion people. If all these women had an HPV screening test every 5 years as recommended, there would be a need for 360 million tests/year, creating the largest demand for any molecular assay in laboratory medicine. This has attracted the interest of the industry, and hundreds of HPV assays are now available on the market. However, most of them have not been tested in clinical trials, and some lack essential performance data [26]. When molecular screening was first introduced, experts outlined some desirable features for any HPV assay intended for CC screening, the VALGENT protocol [25], which, in an updated and more stringent version, indicates only four commercial assays fulfilling the validation requirements. All of them are HPV-DNA assays utilizing a real-time PCR [27], as detailed in Table 1. These criteria are as follows:

o  Tests should preferentially target only the 12 genotypes classified as carcinogenic (HPVs 16, 18, 31, 33, 35, 39, 45, 51, 52, 56, 58 and 59).
o  A sensitivity ≥ 95% for CIN2+ and specificity ≥ 98% for ≤CIN1.
o  The sensitivity and specificity values stated above must be derived from at least three validation studies comparing the test to one of the first-generation assays fulfilling the VALGENT criteria.

Remarkably, these four assays still include HPVs 66 and 68 as targets for screening, since they were developed and validated before the WHO recommended removing these two high-risk HPV genotypes from screening assays, as they reduce the clinical specificity without improving the sensitivity [16]. The scarcity of qualifying assays under this stringent approach also stems from the choice of histological endpoints, making studies relying on cytological assessment not valuable for this purpose.

## 4. Triage Alternatives

**Figure 1:**
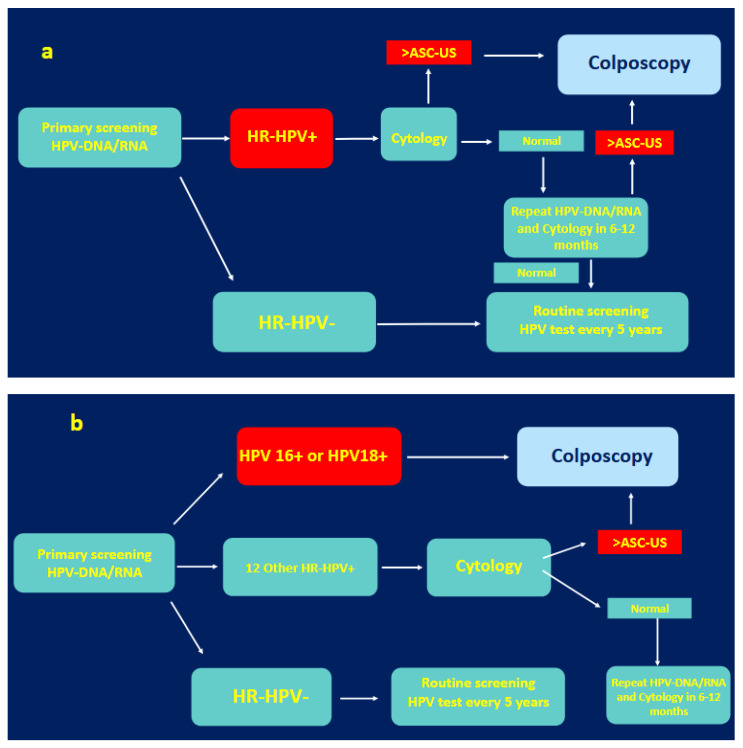
Legend: Flowchart of the 2 most widely adopted triage algorithms for HPV-based CC screening. (**a**)—cytology triage process; (**b**)—HPV genotyping triage process.

Cytology is currently the most used triage method for HPV-DNA/RNA+ samples. Usually, patients showing Atypical Squamous Cells of Undetermined Significance (ASCs-US) or higher-grade results (low-grade squamous intraepithelial lesions, LSILs; Atypical Squamous Cells; cannot exclude a high-grade squamous intraepithelial lesion, ASC-H; high-grade squamous intraepithelial lesions, HSILs) are referred to colposcopy, while those with normal cytology are invited to have their next screening round after a shorter time interval, typically 1–3 years, instead of the regular 5 years for those found to be HPV-negative. Another common algorithm takes into consideration the oncogenic potential of the HPV genotype present in the sample. Patients harboring either HPV 16 or 18 are directly referred for a colposcopy, obviating the need for an intermediary cytology assessment. This direct flow is based on the risk of HPV 16/18 carriers to present a prevalent CIN 2+ which is between 5 and 15% [29]. Figure 1 shows flowcharts for both algorithms. 

### 4.1. HPV Genotyping 

While there are 12 HPV genotypes that are definitely oncogenic and need to be included as targets in any CC screening method, assays may differ in how they identify these 12 HPVs, whether individually or grouped, and in the latter case, what sort of grouping they use. Assays unable to at least individually identify HPVs 16 and 18, like Hybrid Capture II, are clearly outdated, since clinicians and public health authorities require this information. Hence, the standard format of the current CC screening assays is to identify these two highly oncogenic viruses individually and another 12 HPV genotypes grouped into a high-risk non-16/non-18 category. In fact, this group of oncogenic HPVs for which detection is mandatory was, until recently, composed of 14 genotypes: HPVs 16, 18, 31, 33, 35, 39, 45, 51, 52, 56, 58, 66 and 68. As mentioned above, HPVs 66 and 68 were subsequently proven to carry very low or no oncogenic potential; thus, their inclusion in screening assays could lead to a substantial loss of clinical specificity, unnecessary follow-ups and overtreatment. Consequently, their exclusion from molecular screening assays is recommended [27]. The BD Onclarity HPV Assay (BD Diagnostics, Sparks, MD, USA) identifies five genotypes separately (HPV16, HPV18, HPV31, HPV45, HPV52) and another eight genotypes aggregated into three groups: HPV33 and HPV 58; HPV 35, HPV 39 and HPV 68; and HPV 56, HPV 59 and HPV 66. Full genotyping is currently only possible using the Anyplex II HPV HR Detection (Seegene, Seoul, Republic of Korea) assay or its newer version, the Allplex HPV HR assay. The WHO recently published a target product profile document suggesting four groups along a gradient of oncogenicity: 1a HPV 16; 1b HPV 18 and HPV 45; 1c HPV31, HPV33, HPV35, HPV52 and HPV58; and 1d HPV39, HPV51, HPV56 and HPV59 [16]. It is likely that new and existing assays will abide to this new grouping format or identify each of the 12 types individually, which will accordingly allow for the differential management of individuals. A Swedish program evaluated HPV genotyping and clinical follow-up data of thousands of women to propose an improved management algorithm. This allowed for the inclusion of young women starting from the age of 23, while participation in HPV screening programs usually starts at 30 years old, and a more conservative approach to women infected by HPVs of a lower oncogenicity (HPVs 35, 39, 51, 56, 59, 66 and 68) [30]. In the US, recommendations have been developed on how to utilize extended genotyping information in the context of triage [31]. 

### 4.2. DNA Methylation

DNA methylation is an epigenetic mechanism for controlling gene expression. In general, methylated genes are silenced, while non-methylated genes are prone to being expressed. The methylation of growth suppressor genes is an early event in carcinogenesis and has been increasingly explored in cancer diagnostics. In cervical carcinogenesis, several host and viral genes are methylated, as described in Ref. [32]. The use of a methylation-specific PCR to identify these events is being pursued as an alternative to cytology for the triage of HPV+ women [33], and commercial kits with good clinical performance are available on the market for this application [34]. This alternative is particularly useful in situations where cytology cannot be performed, like in the case of self-collected samples. Self-sampling is an important complementary strategy to reach women who do not participate in CC screening programs or have samples regularly collected by health care professionals. If these samples are found to be HPV+, patients need to be invited for cervicovaginal collection at a health establishment, with a high chance of non-attendance. In these cases, if triage can be performed and the results are positive, this has high predictive value for a cervical lesion and actively calling patients in for further tests is justified [35].

### 4.3. Dual Stain

**Figure 2:**
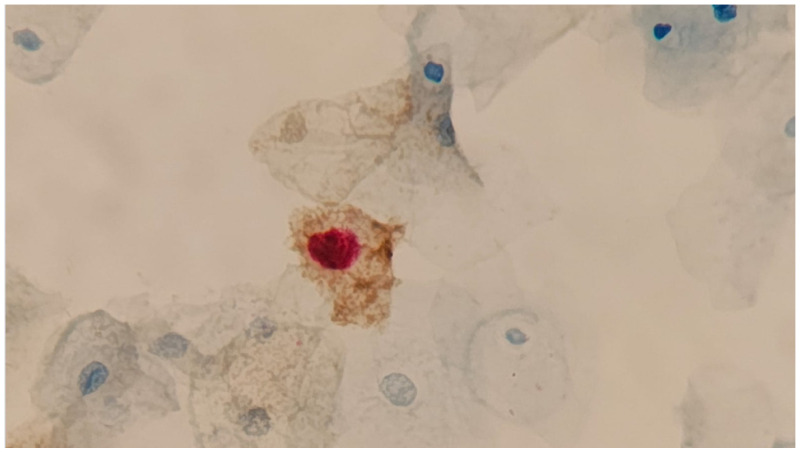
Cervical cells subjected to CINTec Plus immunocytochemistry (Roche Diagnostics, Indianapolis, IN, USA). A dysplastic cell with its nucleus stained in red (Ki-67) and its cytoplasm in brown (p16) is diagnostic of a high-grade lesion. 400X. Courtesy of Dr Cristovam Scapulatempo, Dasa, Brazil.

Here, a dual stain (DS) refers to an immunocytochemistry assay commercially named CINTec Plus™ (Roche Diagnostics Indianapolis, IN, USA ). This assay targets two proteins that interfere with the cell cycle: p16, which normally blocks cell cycle progression and is overexpressed as a response to Rb protein inactivation by the HR-HPV E7 [36], and Ki-67, a nuclear protein that is a proliferation marker for different types of cancer [37]. Each protein is detected with a distinct color and cellular localization; p16 stains on the cytoplasm, whereas Ki-67 in the nucleus. The presence of one or more cells with dual staining is interpreted as being highly predictive of the presence of CIN2+ lesions (Figure 2). The use of DS in different triage scenarios has been thoroughly evaluated and is described in Ref. [38].

### 4.4. Point-of-Care (POC) and New Molecular Assays 

We may separate POC HPV molecular assays into two groups: those using amplification methods and instruments providing all the reagents for extraction, amplification and genotyping in a single cartridge, generally with a very low throughput and high cost, and those relying on isothermal amplification, which may be carried out at room temperature or in simple 37 °C incubators, which have a variable throughput and low cost. Though, in principle, these have been developed for the same purpose, i.e., HPV detection for CC screening, they are adopted in very different settings: the former are used in more affluent environments, and the latter are more appropriate for use in less developed and more remote regions. An example belonging to the first group is the Xpert HPV (Cepheid, Sunnyvale, CA, USA), which has been prequalified for CC screening by the WHO [39]. This assay can be run in only 60 min, identifying HPV 16 in one channel, 18/45 in a separate channel and all 11 other high-risk genotypes (31, 33, 35, 39, 51, 52, 56, 58, 66 and 68) in a third channel. It is very simple to operate and does not require specialized technicians.

The second group is represented by isothermal amplification techniques, such as LAMP (loop-mediated isothermal amplification). LAMP assays were greatly improved and widely deployed during the COVID-19 epidemic. These methods are particularly advantageous for use in regions with few resources as they operate at a constant temperature and do not require complex equipment such as thermocyclers and fluorescence readers. Barra and cols. Ref. [40] developed such an assay targeting HPVs 16, 18 and 45 and challenged it against real-world samples from Mozambique, preserved in an LBC medium, showing excellent concordance with Xpert HPV results [40]. Another example is the ScreenFire HPV RS test (Atila Biosciences Inc., Sunnyvale, CA, USA), which works by subjecting lysates from LBC samples to a single round of isothermal (60 °C) amplification/detection/genotyping. This test identifies 13 high-risk HPV genotypes categorized into different oncogenic risk groups—16 is identified individually, while 18/45, HPVs 31/33/35/52/58 and HPVs 39/51/56/59/68 are identified together. It can be run in only 60 min, and thermocyclers can be used for isothermal incubation, resulting in a high throughput [41]. However, both of the assays described above still require some laboratory infrastructure, preventing their use in some areas of low- and middle-income countries, where a large number of CC cases occur. To address this, several POC assays are in development and undergoing validation using a range of alternative DNA extraction and amplicon detection systems that operate without electrical power, like lateral flow devices, while remaining at an affordable cost. [42] 

## 5. Conclusions

The Nobel Prize of Medicine awarded to Prof. Harald zur Hausen in 2008 paid tribute to his tenacious pursue of the link between HPV and CC [43]. Following the publication of his pioneering work, a generation of scientists and medical professionals has had the privilege of witnessing the identification of the cause of a highly incident public health problem and the development of tools for its resolution. Today, technologies derived from HPV research place CC in a unique position among human cancers, with its elimination achievable in the next few decades. Molecular screening is a key tool we can use to achieve this noble objective, alongside vaccination, and new developments will hopefully counteract the low clinical specificity of HPV testing through the design of innovative triage methods.

## Figures and Tables

**Table 1:** Characteristics of the four assays fulfilling the criteria for 2nd-generation CC screening tests according to Arbyn M et al. [28].

Assay	Manufacturer	Target	Genotyping Coverage
RealTime High-Risk HPV Test	Abbott	HPV L1	HPV types 16 and 18, identified individually, and 31, 33, 35, 39, 45, 51, 52, 56, 58, 59, 66 and 68, aggregated as other HR-HPVs
Onclarity HPV Assay	BD Diagnostics	HPV E6/7	5 types identified individually (HPV16, HPV18, HPV31, HPV45, HPV52) and 3 groups of types aggregated (HPV33/58, 35/39/68 and 56/59/66)
Cobas-x800 HPV Test	Roche Molecular Systems	HPV L1	HPV types 16 and 18, identified individually, and 31, 33, 35, 39, 45, 51, 52, 56, 58, 59, 66 and 68, aggregated with other HR-HPVs
Anyplex II HPV HR Detection	Seegene	HPV L1	HPV types 16, 18, 31, 33, 35, 39, 45, 51, 52, 56, 58, 59, 66 and 68, individually identified
